# Fatal Anaphylaxis to Contrast a Reality: A Case Report

**DOI:** 10.7759/cureus.6214

**Published:** 2019-11-21

**Authors:** Amit Sapra, Priyanka Bhandari, Megha Manek, Supriya Gupta, Shivani Sharma

**Affiliations:** 1 Family and Community Medicine, Southern Illinois University School of Medicine, Springfield, USA

**Keywords:** nonionic contrast, osmolality, antibody, histamine, anaphylaxis, hypersensitivity

## Abstract

We are describing the case of a 45-year-old female with a past medical history of severe chronic obstructive pulmonary disease (COPD), type 2 diabetes mellitus, and anxiety and with no known allergies to contrast media. The patient presented to her primary care doctor’s office with typical symptoms of COPD exacerbation. She was given a five-day course of prednisone (40 mg/day) and Azithromycin and advised to follow up with her pulmonologist. The patient called her pulmonologist’s office five days later due to non-relief of symptoms and was advised to get a chest radiograph. The chest X-ray did not show evidence of any acute changes. Her symptoms continued to worsen, and she was advised to get a computerized tomography (CT) of the chest with pulmonary embolism (PE) protocol, where 60 ml of Isovue-370 (Iopamidol - a non-ionic radiocontrast dye) was injected per the PE protocol. She had an unpredictable fatal anaphylactic reaction to non-ionic contrast dyes and suffered a cardiac arrest while getting the scan done.

## Introduction

Contrast media (CM) are widely used in imaging techniques to enhance the differences between body tissues on images. Less than one percent of patients receiving low osmolar nonionic contrast media can develop anaphylaxis, including a severe anaphylactic shock. The precise mechanism of this is mostly unknown but postulated to be due to the release of histamine by triggering mast cells or IgE-related mechanisms. The unpredictability of a negative past medical history of adverse reactions to these dyes and the considerable variability in the pretreatment regimens for patients with the previous adverse response to these dyes further confounds the whole picture [1]. As primary care providers, we are usually the first link of the patient to healthcare access. Therefore, we feel the great need to generate awareness of this rare but life-threatening emergent condition and be well prepared to deal with it.

## Case presentation

**Figure 1:**
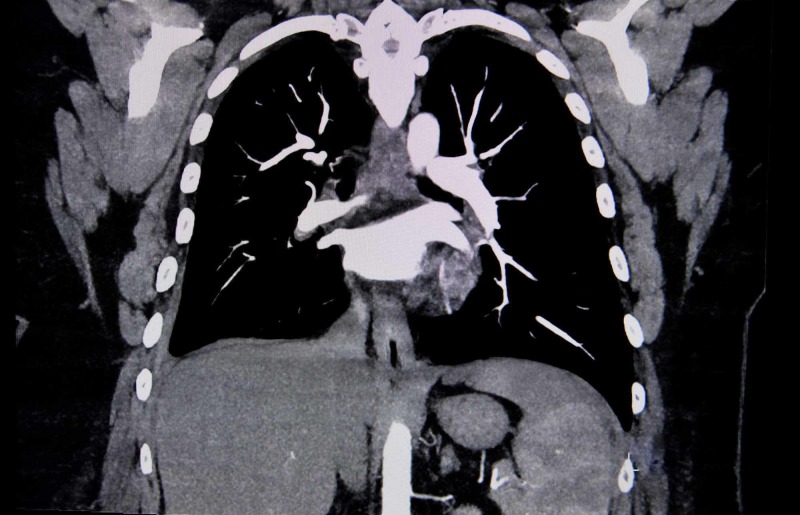
Computerized tomography of the chest of the patient did not show any evidence of pulmonary embolism.

We describe the case of a 45-year-old female with a past medical history of severe chronic obstructive pulmonary disease (COPD), type 2 diabetes mellitus, anxiety, glaucoma, and no known contrast allergies. She had received contrast dye during imaging done in the past without any adverse reactions. She was recently evaluated in our family medicine clinic for another episode of her COPD exacerbation despite being on her controller inhalers. She received a five-day course of oral prednisone (40 mg/day) and azithromycin and was advised to follow up with her pulmonologist. The patient called her pulmonologist’s office five days later due to non-relief of symptoms and was advised to get a chest X-ray. Her chest X-ray showed no evidence of any acute changes, but the patient continued to have worsening shortness of breath. She again called her pulmonologist’s office and was advised to get a CT chest with pulmonary embolism (PE) protocol (Figure 1).

The next day she underwent a CT chest with PE protocol where 60 ml of Isovue-370 (Iopamidol) - a nonionic radiocontrast dye - was injected per the PE protocol. Within minutes of inserting the dye for the scan, the patient became dyspneic and hypoxic, unresponsive, and pulseless. Immediate cardiopulmonary resuscitation (CPR) was started, and she received two rounds of intravenous (IV) epinephrine and was started on the bag and mask ventilation with oxygen was initiated. Emergency medical services (EMS) arrived, and the airway was secured with a king airway. An 18-gauge intravenous line placed, and she was transferred to the emergency department (ED). In the ED, she was found to be unresponsive, hypotensive, had fixed dilated pupils, and was experiencing severe respiratory distress.

She then developed apneic episodes and had a Glasgow Coma Scale of 3. Advanced cardiac life support was initiated. There was no shockable rhythm detected. She received a total of five ampules of epinephrine, two ampules of bicarbonate, IV magnesium, IV Benadryl methylprednisone, and finally, a norepinephrine drip was started which led to return of spontaneous circulation (ROSC). By this time, her imaging report came back and was negative for any pulmonary embolism.

**Figure 2:**
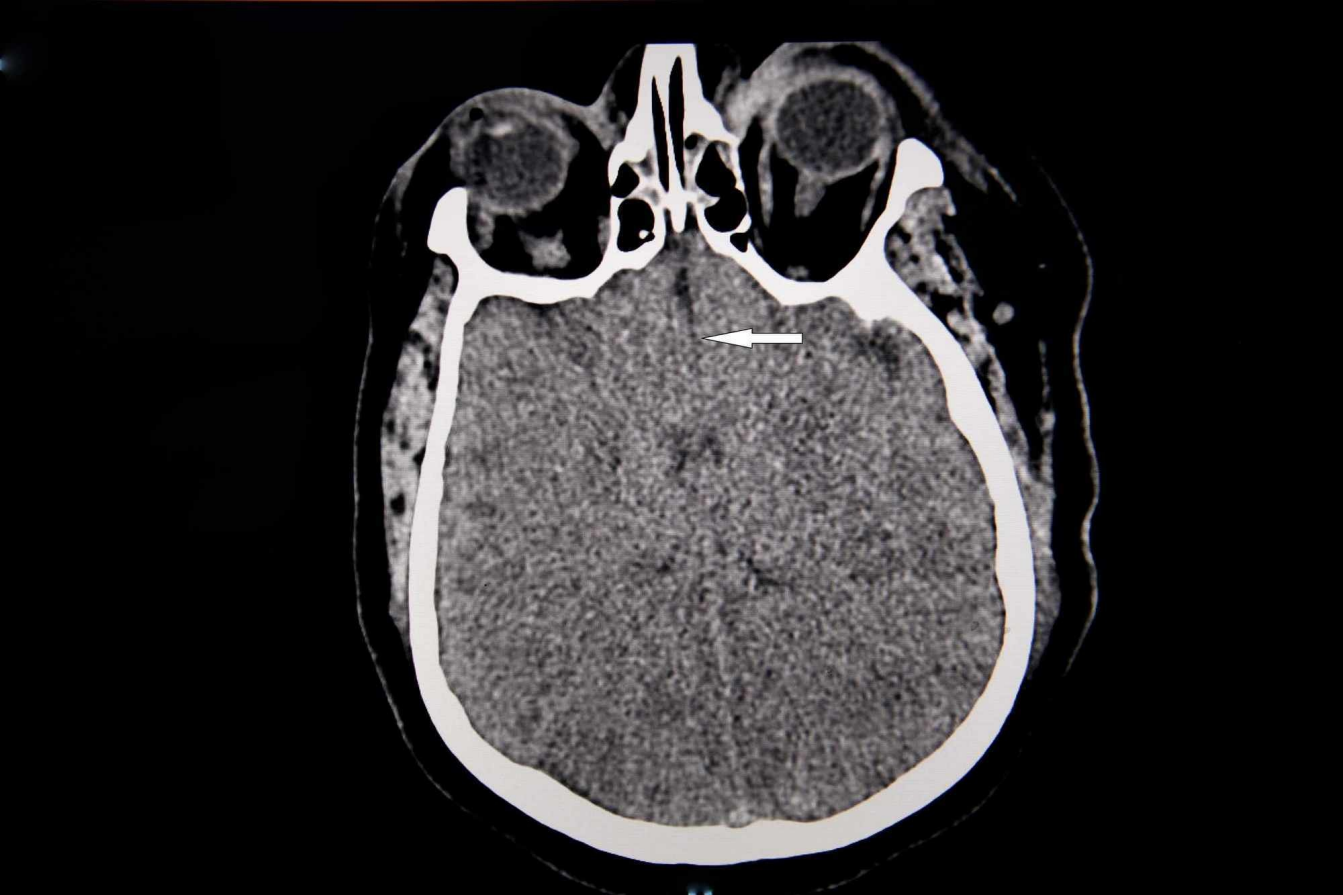
Computerized tomography of the brain of the patient showing diffuse effacement of the sulci.

The patient was subsequently moved to the intensive care unit, where she was switched to mechanical ventilation. She was also found to have refractory bronchospasm. She required excessive positive end-expiratory pressure (PEEP) to as high as 22-24 centimeters of water, ventilator settings were manipulated to maximize expiratory time, and the respiratory rate and tidal volume were decreased. She also continued to receive methyl-prednisone, bronchodilators, and Benadryl. The septic screen was sent, and she was empirically started on IV vancomycin and IV piperacillin-tazobactam plus received IV sodium acetate for acidosis. Her toxicology screen came back as negative. Echocardiogram was done, which showed preserved left ventricular ejection fraction. The patient’s electroencephalogram showed profound generalized cerebral dysfunction with absent posterior dominant rhythm (PDR) suggestive of anoxic brain injury. Computerized tomography head reported diffuse cerebral anoxia (Figure 2), and the patient was declared brain dead the next day. The family refused autopsy.

## Discussion

Anaphylaxis is regarded as the most dangerous form of an allergic reaction with the potential of causing fatal consequences. Experts from the World Allergy Organization (WAO) define anaphylaxis as “a severe, life-threatening generalized or systemic hypersensitivity reaction” [2]. Anaphylaxis can be IgE-mediated or non-IgE mediated. When faced with such a life-threatening emergency, it is not possible to determine the mechanism that is causing it. The etiology of most immediate hypersensitivity contrast reactions is not entirely understood.

The classic hypothesis around IgE-mediated anaphylaxis is that the patient has to be sensitized towards the allergen before the full-blown reaction [2]. Administration of the dye several times possibly causes this sensitization, which could have possibly happened in our case as well [3]. Studies have reported that, in more than 90% of cases, the direct release of histamine and other mediators was responsible for the anaphylaxis symptoms after administration of non-ionic contrast material, and an IgE-mediated contrast material allergic reaction was identified in the minority of patients. This could likely explain why patients who have never been exposed to contrast media can experience an anaphylactic reaction on first exposure. It reinforces the fact that sensitization is not required for a contrast reaction to occur. Sodium iodide has been used as a contrast medium began in clinical practice as early as the 1920s. Due to high toxicity and weak radiographic enhancement, its use was limited as a contrast medium. In the 1950s, water-soluble sodium and meglumine salt derived from tri-iodinated benzoic acid significantly increased the use of contrast agents. These preparations were less toxic than earlier preparations but were hyperosmolar (up to osmolality five to eight times that of blood). By the 1970s, low-osmolality iodinated contrast media had been developed. High-osmolar and low-osmolar contrast media have led to their being some of the most widely used drugs in the history of medicine [4,5]. Each year approximately 70 million people worldwide receive IV iodinated contrast agents [1,2]. Iodinated contrast media are the most common IV pharmacologic agents of any type currently in use [6,7]. Radio-contrast material is generally well tolerated by the majority of the patient population, although approximately 1% of patients who receive non-ionic contrast media might develop anaphylaxis, including anaphylactic shock. Side effects profiles are guided by the characteristic properties of contrast material such as osmolality, iconicity, and viscosity. The risk of fatal adverse reactions due to iodinated contrast media has decreased owing to the development of ionic, high osmolality to non-ionic, low osmolality contrast media. Katayama et al. reported that the incidence of adverse reactions with iodinated contrast was 12.66% for ionic and 3.13% for nonionic contrast media [8]. Nevertheless, adverse reactions of nonionic contrast media still occur, and some physicians reported that patients exposed to nonionic contrast had higher incidences of severe adverse reactions [9]. Fatal adverse reactions can occur in 0.04% to 0.22% of cases [10].

CT chest with PE protocol is a frequent order that is placed by practitioners, whether working in the clinic, ER, or in the hospitals, to rule out pulmonary embolism. Our patient had also previously undergone CT scans with Iopamidol on multiple occasions but had never experienced any adverse reactions to and hence was also not in her allergy list. Hemodynamic instability due to dye could have also happened due to coronary spasm, for which nitroglycerine is usually administered. In our case, the severe clinical bronchospasm and lack of evidence of ischemia pointed more towards an anaphylactic picture. A limitation of our case report is the lack of intradermal skin tests for the contrast medium and serum immunologic tests and the refusal of the Power of Autonomy for an autopsy.

## Conclusions

This case gives us insight into the fact that every patient who undergoes a contrast imaging runs a risk of suffering an adverse effect, including a life-threatening anaphylactic shock. This can happen even in patients who successfully underwent imaging in the past without any adversity and have no known contrast allergies. The ordering physician should include a proper assessment of the patient's risk versus potential benefit, available imaging alternatives as well as the presence of a valid clinical indication for the contrast medium administration. Patient selection becomes extremely prudent when we consider those previous allergic reactions to the same class of contrast medium the most significant risk factor for predicting future adverse events. Patients who have had such prior hypersensitivity reactions to contrast medium have an approximately five-fold increased risk of developing a similar reaction when re-exposed to the same class of contrast medium again. Certain factors like history of asthma or usage of beta-blockers increase the likelihood of an allergic-like contrast reaction among the general population. Unfortunately, pretesting with intradermal skin tests with contrast media is not found to be useful in minimizing reaction risk.
